# Differential loss of components of traditional ecological knowledge following a primate extinction event

**DOI:** 10.1098/rsos.172352

**Published:** 2018-06-13

**Authors:** Samuel T. Turvey, Jessica V. Bryant, Katherine A. McClune

**Affiliations:** 1 Institute of Zoology, Zoological Society of London, Regent's Park, London NW1 4RY, UK; 2 Department of English, University of Bristol, 3-5 Woodland Road, Clifton, Bristol BS8 1TB, UK

**Keywords:** biocultural diversity, China, folktales, Hainan gibbon, indigenous knowledge, oral tradition

## Abstract

Traditional ecological knowledge (TEK), an important component of the modern conservation toolkit, is being eroded in indigenous communities around the world. However, the dynamics of TEK loss in response to ecosystem change and disruption to social–ecological systems, and patterns of variation in vulnerability and resilience of different components of TEK, remain poorly understood. The Hainan gibbon (*Nomascus hainanus*), a culturally significant primate, was formerly distributed across Hainan Island, China, but became extinct across most of this range within living memory and is now restricted to a single landscape, Bawangling National Nature Reserve. Gibbon-specific TEK (including folktales, natural history information and methods of gibbon exploitation) is still present in indigenous communities across seven Hainanese landscapes, but statistically significant differences in TEK content exist between landscapes with different histories of gibbon persistence: respondents from Bawangling and most landscapes that have recently lost gibbons report more gibbon-related folktales compared with landscapes from which gibbons have been absent for several decades. Species-specific folktales might have been lost more rapidly compared with other components of TEK because older community members are typically the ‘cultural repositories’ of stories, whereas knowledge about practical interactions with biodiversity might be shared more widely with younger community members.

## 1. Introduction

Most traditional (indigenous and/or rural) communities around the world possess an extremely rich body of knowledge about local environmental resources and biodiversity, which has developed through their interactions with the non-human world around them [1,2]. Different anthropological frameworks exist for classifying indigenous knowledge (e.g. as indigenous and local knowledge, or ILK) [3,4]. However, this body of knowledge can typically be subdivided into two broad categories: (i) local ecological knowledge (LEK), representing experiential knowledge derived from individuals' lived interactions with the local environment, and (ii) traditional ecological knowledge (TEK), the cumulative body of knowledge, beliefs, values and traditions about the natural world that is passed down between generations through oral cultural transmission, and which typically represents the summation of centuries or millennia of ecological adaptation by human groups to their environments [5,6]. Both of these domains of indigenous knowledge are increasingly recognized as constituting invaluable tools to aid in conservation of biodiversity, and TEK in particular is often able to support sustainable use of environmental resources and build resilience in social–ecological systems [6,7], for example by providing practical models for sustainable management [8], or social methods of supporting or enforcing biodiversity preservation based on indigenous value systems [9–12] and indigenous storytelling [13].

TEK represents an inherently dynamic body of information that is continuously updated with successive generations [6]. However, in addition to this ongoing long-term change, the TEK of local communities around the world is becoming eroded and decreasing in overall content. The dynamics and drivers of TEK loss are complex and multidimensional. The process is associated with globalization and exposure of indigenous peoples to Western cultural and economic norms, which has led to widespread loss of local languages and belief systems, and disruption of traditional social–ecological systems as local communities are assimilated into market economies; these major socio-cultural changes are all typically associated with changing patterns and levels of intergenerational communication [7,14–17]. A further important contributing factor to erosion of TEK is the global-scale biodiversity crisis, which is resulting in the progressive defaunation and degradation of ecosystems and the loss of essential services they provide to local communities; this process erodes the non-human landscape from which TEK can be derived and increases the likelihood of disruption to cultural continuity in communities with subsistence economies [1].

As TEK is an important component of the modern conservation toolkit, determining the rate and pattern of TEK loss from local communities, and the fragility or resilience of different components of TEK, constitutes an important conservation research concern to be investigated alongside increased efforts to document and maintain this invaluable body of knowledge. Available case studies demonstrate substantial differences in the retention or loss of indigenous knowledge following ecosystem change. At one extreme, Turvey *et al*. [18] showed a rapid loss of awareness of recently extinct Yangtze megafauna in Chinese fishing communities, when these culturally or economically significant species had disappeared only a few years earlier and during the lifetime of all informants in the study. Conversely, local traditions describing animals that have been proposed to represent species that became extinct centuries or even millennia earlier are reported from numerous cultures [19–23]. Memories of environmental change have been shown to persist under some circumstances for over 7000 years [24], and animal-related and other folktales also appear to be able to survive through oral transmission for millennia [25,26]. However, few comparative analyses have yet been conducted to investigate patterns of vulnerability or resilience to erosion across different components of TEK, to enable evidence-based predictions to be made between cultural systems or to understand the relationship between TEK loss and the status of the non-human environment within which indigenous cultures exist.

Gibbons are long-armed, tailless hylobatid primates that are highly adapted for living in the forest canopy rather than on the ground, and that exhibit a suite of characteristic behaviours including brachiating locomotion and extensive species-specific vocalizations [27]. Gibbons were formerly distributed across southern and central China and have represented culturally significant animals for much of Chinese history, often being assigned supernatural or mythic properties, and with their distinctive song symbolizing the melancholy of travellers far from home in traditional literature [28–31]. However, gibbons have been extirpated across most of China during recent centuries through a combination of habitat loss and hunting for traditional medicine [31], and one Chinese species, the Hainan gibbon (*Nomascus hainanus*), is now probably the world's rarest mammal [32–34]. During the twentieth century, the Hainan gibbon was still distributed across much of the forested mountainous interior of Hainan Island, China's southernmost province [32], and cultural practices and traditions associated with the species in Hainan's indigenous Li and Miao ethnic communities are recorded in Imperial-era and Nationalist-era local *difangzhi* gazetteers [35] (table 1), although these have never been systematically collected and described. The species now survives only as a remnant population of approximately 26 known individuals within a single protected area, Bawangling National Nature Reserve [32–34], and the approximate timing of its disappearance from other protected areas across Hainan has been estimated through field surveys and analysis of patterns of local last-sighting records [32,36–38]. A second native primate, the rhesus macaque (*Macaca mulatta*), also occurs on Hainan and is still relatively common across the island [39,40].

**Table 1.** Cultural practices and traditions about gibbons recorded from Hainan in historical *difangzhi* gazetteers [35].

description	reported date
(*a*) fantastic information on gibbon natural history	
good at climbing but unable to walk; people who raise/domesticate gibbons must keep them in the trees (however, people who raise them sometimes put them on the ground, and they can walk better than monkeys); if they ever fall to the ground or touch the ground they stiffen up like a tree; if they get too close to the vapours of the earth they fall ill and die (but can be revived by drinking boiled monkshood juice)	1774, 1828, 1855, 1877, 1908, 1911, 1917, 1935
some gibbons (‘same-length-armed apes’, *tongbiyuan*) have arms that are connected together at the shoulders, so that when the left arm is stretched the right contracts and when the right arm is stretched the left contracts	1864, 1908, 1931
‘stone apes’ are the size of a fist but grow when fed water (sometimes specifically ‘from a well’); also known as black apes because they can draw using ink, and will jump into the inkpot when they have finished (NB: this tradition sometimes specifically refers to monkeys rather than gibbons/apes)	1774, 1783, 1855, 1864, 1917, 1919, 1935
male otters mate with female gibbons to produce short-clawed otters	1774, 1792, 1855, 1908, 1917, 1931, 1935
gibbons make spicy wine in caves in the cliffs, using rice mixed with flowers; in one cave there would always be 5 or 6 litres of it	1783
(*b*) practical/utilitarian knowledge	
when gibbons and monkeys are observed tossing wild lychees about, this means the fruit are sweet and edible and no longer sour	1935
can be hunted by chasing them to the tips of trees, cutting away the surrounding bamboo, then drawing in a net to catch them	1783
local people use gibbon bones as chopsticks, which can test for poison	1908, 1931

Hainan therefore consists of a series of landscapes containing indigenous communities that are likely to possess extensive TEK on locally occurring biodiversity, and across which there is variation both in local presence or absence of a critically endangered primate species that is known to be culturally significant, and in the amount of time since this species was last locally present in areas where it is now extirpated. Detailed investigation of content and variation of TEK about gibbons across Hainan might therefore serve both to generate new tools to support ongoing conservation efforts for this highly threatened species [33], and also to provide a framework for assessing how different components of TEK persist or erode over time following the loss of culturally relevant biodiversity. As part of our wider-scale ongoing conservation research programme on the Hainan gibbon [33,34], we therefore collected, categorized and analysed a new large-scale dataset of TEK on gibbons from indigenous communities across Hainan, and we demonstrate distinctive and unexpected patterns in TEK erosion following local gibbon extinction that have wider implications for the use of indigenous knowledge in conservation.

## 2. Material and methods

### 2.1. Interview survey

**Figure 1.**
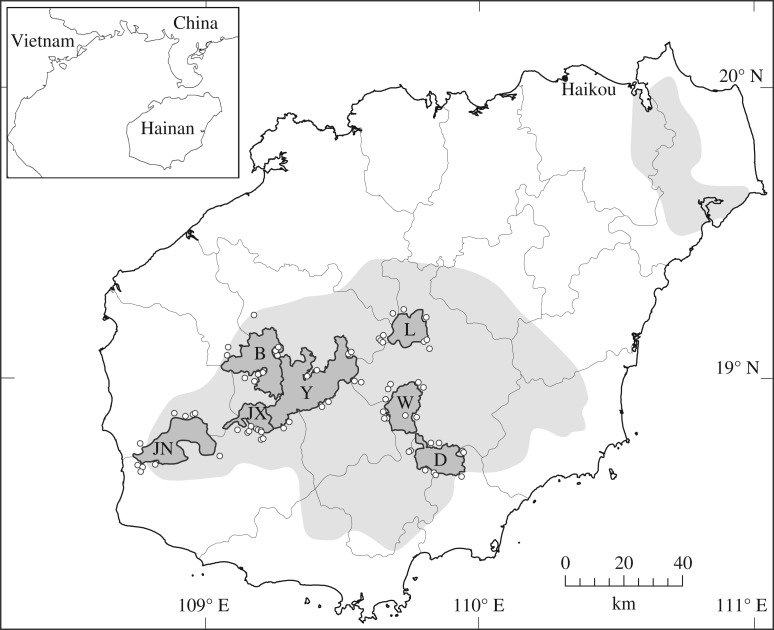
Locations of protected area landscapes across Hainan at which interviews were conducted, showing locations of surveyed villages (circles) and inferred Hainan gibbon distribution in 1900 (pale grey), after Chan *et al.* [32]. B, Bawangling; D, Diaoluoshan; JN, Jianfengling; JX, Jiaxi; L, Limushan; W, Wuzhishan; Y, Yinggeling.

We conducted interviews in January and April 2015 in communities around seven protected area landscapes in Hainan: Bawangling, Diaoluoshan, Jianfengling, Wuzhishan and Yinggeling National Nature Reserves, and Jiaxi and Limushan Provincial Nature Reserves (figure 1). Of these, Bawangling contains the only surviving Hainan gibbon population, and now-extirpated gibbon populations were also present in the other six landscapes within living memory [36–38]. The precise date of local gibbon extirpation in each landscape is not fully understood, but gibbons appear to have persisted in some landscapes much more recently than in others, with available data providing a broadly consistent pattern of the relative timing of disappearance from different areas. Based on local surveys conducted on Hainan up to the early 1980s that likely did not cover all of these landscapes, Liu *et al*. [36] considered that by this point gibbons had already disappeared from Jianfengling and Wuzhishan, but still survived in Bawangling, Limushan and Yinggeling. Using later information from hunters, government and reserve agencies and other local records that again was not available for all landscapes, Zhou *et al*. [37] suggested that gibbons disappeared from Diaoluoshan, Jianfengling and Wuzhishan during the 1980s, but persisted in Limushan into the 1990s. Previous analysis of the extensive LEK dataset for gibbons obtained from these landscapes during our survey [38] demonstrated statistically significant differences between Bawangling versus Diaoluoshan, Jianfengling and Wuzhishan across multiple indices of respondent awareness, experience and sighting histories, providing strong and reasonably consistent support for probable local extinction of gibbons several decades earlier; conversely, LEK data for Jiaxi, Limushan and Yinggeling showed few statistical differences from Bawangling in awareness, experience or sighting histories of gibbons by local respondents, and relatively recent detailed sightings were collected from these reserves, suggesting that gibbons had probably become locally extinct much more recently.

People are not allowed to live inside the reserves, but numerous villages are located close to the boundaries of each reserve, and local people use animal and plant resources collected from inside the protected areas [32,33,39,41]. We randomly selected 10 villages around each reserve in which to conduct interviews, from a full list of all local villages provided by the respective reserve management office at Bawangling, Diaoluoshan, Jianfengling, Jiaxi and Wuzhishan, and from a list of the subset of villages close to specific areas of the reserve where relatively recent gibbon reports had originated at Limushan and Yinggeling [38]. We aimed to conduct a target number of 10 interviews per village to comply with predicted response saturation levels and capture existing variation in responses [42]. Villages around reserves are all relatively small (data for eight villages around Bawangling: village population = 113–710 inhabitants and 25–138 households [41]). A detailed plan of households was not available, so we selected most respondents by walking through each village and encountering people at random, typically traversing the entirety of the villages in the process. We also included a small amount of targeted ‘snowball sampling' [2], as local village heads sometimes introduced us to respondents they considered to be knowledgeable about local wildlife. We interviewed respondents of both sexes and any reported occupation. We did not interview children and teenagers below the age of 18, and we only interviewed one respondent per household to ensure independence of responses.

We used a standard anonymous questionnaire for all interviews, which took up to 1 h to complete (electronic supplementary material, S1). Interviews were mainly conducted in Mandarin or Hainanese, and recorded in Chinese, by pairs of volunteers recruited from universities or NGOs in Hainan; most local people could understand and communicate in these languages, although other local ethnic minority languages (Li/Hlai, Miao/Hmong) were also relatively widely spoken in target communities. The four-person team of interviewers changed between January and April 2015 except for one team member, who led the second survey period to ensure consistency in interview methods. We assured all respondents of confidentiality and that they could end interviews at any time, briefed them on the objectives of our research and how interview data would be stored, and obtained their consent prior to carrying out interviews.

The main purpose of our survey was to collect LEK data on local occurrence and status of nine mammal species: wild pig (*Sus scrofa*; 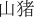 *shanzhu*), rhesus macaque (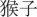 *houzi*/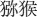 *mihou*), Hainan gibbon (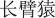 *changbiyuan*), clouded leopard (*Neofelis nebulosa*; 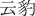 *yunbao*), Asian black bear (*Ursus thibetanus*; 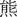 *xiong*), Chinese pangolin (*Manis pentadactyla*; 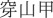 *chuanshanjia*), binturong (*Arctictis binturong*; 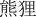 *xiongli*), sambar deer (*Rusa unicolor*; 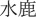 *shuilu*) and giant anteater (*Myrmecophaga tridactyla*; 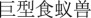 *juxingshiyishou*). Most of these species are known or suspected to occur in Hainan [40,43]; giant anteaters, native to Central and South America, were a negative control to check response accuracy [44,45]. Incorporation of a range of species was intended to obscure the potential importance to interviewers of any single species, and therefore increase the likelihood of respondents reporting potentially sensitive information about gibbons [46].

We first collected demographic data from respondents on their age, sex, ethnicity, primary occupation, how regularly they reported visiting local forests, and how long they had lived in the community where they were interviewed. We then showed respondents colour photographs of the nine mammals and asked them to name each species. We sourced photographs from www.arkive.org and the Zoological Society of London, and showed them in the same order (as listed above) in all interviews. After showing each photograph, we asked respondents to provide further ecological and morphological details to confirm accurate species recognition. If they did not recognize species from photographs, we used standard Chinese names to prompt recall. We asked respondents a series of questions related to their LEK of each species; analyses of these data and their implications for conservation are reported in [38,47]. When discussing gibbons, we also asked an additional open-ended question about TEK: ‘Have you heard any stories about gibbons or anything else about them, such as uses?' (‘
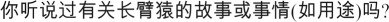
’) We asked this question using the same wording and at the same place in the questionnaire in all interviews (electronic supplementary material, S1).

### 2.2. Analysis

We recorded whether respondents answered our open-ended gibbon TEK question, and grouped respondents' responses to the question into different categories based on the content of their response. We then analysed the TEK dataset using R v. 3.2.3 [48], to investigate whether variation in (i) respondent TEK retention and (ii) respondent TEK content was determined by variation in interview locality (reserve) using multiple regression.

Nearly all respondents (89%) had always lived in their local village, so we did not include the low variation associated with this demographic parameter in our analyses. Conversely, there are statistically significant differences in proportions of sexes, ethnic groups and people reporting that they visit the forest regularly, represented in respondent samples between reserves [38]. To control for this respondent variation, we conducted multiple regression using generalized linear models (GLMs) to investigate five of the six demographic variables and interview locality (reserve) as fixed effects, using Gaussian error structure (age) or binomial error structure (sex, ethnicity, frequency of forest visits, occupation) and logit-link function. We first modelled each variable separately, and included all significant variables in each final full additive multivariate model. We then applied a hypothesis-testing approach using stepwise model selection, deleting the non-significant predictor variable with the highest *p*-value at each step and model-checking to assess subsequent significance of changes in deviance resulting from removal of terms [49]. We analysed the entire respondent dataset when investigating variation in TEK retention, and analysed data for the subset of respondents who reported TEK when investigating variation in TEK content. Respondents reported belonging to four ethnic groups (Han, Li, Miao, Zhuang), but due to low occurrence of Zhuang respondents (*n* = 2), we considered only respondents belonging to Han, Li and Miao ethnic groups when analysing the influence of ethnicity on responses. We assigned occupations to two categories: ‘forest-related jobs' (9%, including rubber harvesters, loggers, and reserve and forestry employees) and ‘non forest-related jobs' (91%; mainly comprising farmers, 84%). We also assigned data on frequency of forest visits to two categories, more than once per month (29%) and less than once per month (71%), to reflect a natural break in the data. Variation in respondent responses between reserves was compared against data for Bawangling, as this is the only landscape that still contains gibbons and so is considered least likely to have experienced erosion of gibbon-specific TEK.

## 3. Results

We interviewed 709 respondents (Bawangling, *n* = 107; Diaoluoshan, *n* = 100; Jianfengling, *n* = 100; Jiaxi, *n* = 101; Limushan, *n* = 100; Wuzhishan, *n* = 100; Yinggeling, *n* = 101; mean age = 50.1, age range = 20–94 years, s.d. = 15.3; male = 83%, female = 17%; Li = 84%, Miao = 11%, Han = 4%, Zhuang < 1%). Overall, 54% of respondents could identify a gibbon photograph correctly, and 49% were familiar with the standard Chinese name for gibbon; respondents used numerous names when discussing gibbons, including words in different minority languages [38]. Of our respondent sample, 99 respondents (14%) volunteered specific relevant information about gibbon TEK, with most respondents only providing a single piece of information (electronic supplementary material, table S1). A small number of additional non-relevant responses to the question (e.g. ‘I've seen them on TV', or details of encounters with gibbons) were discarded from analysis. Gibbon TEK was reported from all seven study landscapes.

Gibbon TEK reported by different respondents fell into five distinct categories: (i) accurate information on gibbon ecology, behaviour or other aspects of natural history (*n* = 37); (ii) inaccurate (or irrelevant) information on gibbon ecology, behaviour or other aspects of natural history (*n* = 26); (iii) practical/utilitarian knowledge about cultural usage of gibbons (*n* = 28); (iv) cultural values/attitudes relating to gibbons (*n* = 6); (v) folktales about gibbons (*n* = 38) (table 2). Almost none of this newly collected TEK matches historical cultural practices and traditions about gibbons recorded from Hainan in Imperial-era and Nationalist-era local *difangzhi* gazetteers; the only themes that are shared between these two bodies of knowledge are the ecologically inaccurate idea that gibbons will die if they come down to the ground, and the reported use of gibbon bones as chopsticks (table 1). Six different gibbon folktale categories were reported (table 3). No exact homologues to these folktales exist in previous Chinese ethnographic studies, although our folktale type 3 (gibbons capture people to eat) and the related ‘fear of being eaten by gibbons' (table 2) may both be related to Chinese animal tale 312A* in Ting [50], where a girl is threatened with being eaten by a monkey.

**Table 2.** Five distinct categories of TEK about gibbons reported from respondents living close to forested areas in Hainan.

TEK category	details
accurate information on gibbon natural history	climb trees/live in trees; do not come down from trees; flee when they encounter humans; make loud melodic calls; call in the morning; call every day; hold their offspring tightly; arms much longer than legs; eat sweet fruit; there are dark and pale colour morphs
inaccurate/irrelevant information on gibbon natural history	too lazy to look after their young; monogamous; love to fight, especially with monkeys; like people; like to smile and laugh; call in the afternoon or evening; search for ropes to climb; will come down from trees; only come down from trees if leaves are covering the ground; die if come into contact with ground; when they die in the trees, hold onto branches/companions carry dead individual away/companions have to bite through branches to release dead individual; steal yams; eat corn; drink water from holes in trees; hold hands and work in relays to drink water or climb trees; play in water
practical/utilitarian knowledge about cultural usage of gibbons	(i) Hunting: difficult to catch/shoot; have to cover ground with leaves otherwise gibbon will not fall out of tree, and will still hang onto branches even if shot
	(ii) Usage: good to eat; can be used as ingredient in medicine; bones (always arm bones if specifically described) keep person safe if carried; bones can be used as medicine (or pesticide), to make chopsticks, to test for poison, or to make chopsticks specifically used to test for poison (bubbles come out of bones; food will move, ‘react’, ‘gush’ or explode)
cultural values/attitudes relating to gibbons	unlucky to see; people are afraid of being captured or eaten by gibbons; should be worshipped; do not shoot them, because people were saved by gibbons in the past
folktales about gibbons	see table 3

**Table 3.** Six distinct gibbon folktale types reported from respondents living close to forested areas in Hainan.

folktale type	details/folktale subtypes
1. People turned into gibbons (*n* = 27; only 14 respondents reported story details)	(A) children with nothing to eat go into mountains to find food (fruit) and turn into gibbons who do not come back down from trees; sometimes described as being orphans, or driven by wicked stepmother (who does not give them food, sends them to forage for food in mountains, gives them wood or faeces to eat, does not want children so sends them to mountains to get rid of them, or sends them to guard crops which are then eaten by birds so she withholds their food as punishment); sometimes their father wants them to come back to hug them but they refuse, or tries to persuade them to return home by pretending to be dead (*n* = 6)
	(B) lazy or untalented people (e.g. do not know how to weave Li shirts), sometimes specifically referred to as ‘primitive humans’, exhibit behaviours leading them to turn into gibbons, such as want food without having to work, so pick fruits, making them climb trees, their arms grow longer, and they grow hair; run into the mountains, or go to mountains to pick fruit to eat; specifically told to go and be a gibbon if they do not want to work; steal corn or squash*; burned on backside to make it red*; make clothes out of fibres and tail out of cotton*; sometimes confronted by monkeys for appearing different, so made a fake tail out of squash (* indicates stories which appear to refer to monkeys instead of gibbons, but which the respondent specifically said related to gibbons) (*n* = 7)
	(C) before Communist Liberation of Hainan (in 1950), no-one cut their hair so they turned into gibbons (*n* = 1)
2. Gibbons turned into people (*n* = 7)	always very briefly explained, in terms of gibbons being ‘ancestors’
3. Gibbons capture people to eat (*n* = 1)	no further details provided
4. Gibbons have helped/saved people (*n* = 4)	no further details provided
5. Gibbons cannot have more than 10 children, or they chase extra children away from home (*n* = 1)	specifically described by respondent as ancient story rather than natural history description
6. Gibbons unable to come down from trees (*n* = 2)	gibbon makes bet with another animal (either monkey or earthworm) about being able to come down to ground and other animal being able to climb tree; results in gibbon being killed or going blind if it ventures down to ground; sometimes other animal also dies if it climbs tree

We investigated demographic and spatial patterns of variation in all of these primary TEK categories except for cultural values/attitudes, as the sample size for this category was too small to permit meaningful statistical investigation (table 4). The only significant predictor of whether respondents reported gibbon TEK was age, with older respondents more likely to be aware of some component of TEK (s.e. = 0.001, *z* = 5.300, *p* < 0.0001; mean age of respondents reporting TEK = 57.6 years, mean age of respondents not reporting TEK = 49.0 years), and with only three respondents younger than 30 (6.5% of this age category) reporting any TEK (two young respondents reported practical/utilitarian knowledge and one reported a folktale; electronic supplementary material, table S1).

**Table 4.** Final models investigating variation in TEK about gibbons across Hainan, with reserve data compared against data for Bawangling. Significant results highlighted in italics.

predictor	estimate	s.e.	*z*-value	*p*-value
1. reporting any TEK				
intercept	−0.082	0.044	−1.881	0.060
age	0.004	0.001	5.300	<*0*.*0001*
2. reporting accurate natural history				
intercept	−2.565	1.038	−2.472	*0*.*013*
reserve (Diaoluoshan)	2.698	1.160	2.327	*0*.*020*
reserve (Jianfengling)	2.853	1.289	2.214	*0*.*027*
reserve (Jiaxi)	−16.001	1809.055	−0.009	0.993
reserve (Limushan)	2.342	1.141	2.052	*0*.*040*
reserve (Wuzhishan)	3.007	1.122	2.679	*0*.*007*
reserve (Yinggeling)	1.312	1.311	1.001	0.317
3. reporting folktales				
intercept	2.565	1.038	2.472	*0*.*013*
reserve (Diaoluoshan)	−3.577	1.191	−3.004	*0*.*003*
reserve (Jianfengling)	−4.357	1.498	−2.909	*0*.*004*
reserve (Jiaxi)	−0.080	1.470	−0.054	0.957
reserve (Limushan)	−21.131	1537.401	−0.014	0.989
reserve (Wuzhishan)	−5.656	1.457	−3.882	*0*.*0001*
reserve (Yinggeling)	−1.312	1.311	−1.001	0.317

Within the subset of respondents who reported gibbon TEK, none of our chosen variables predicted likelihood of reporting either practical/utilitarian knowledge or inaccurate information about natural history, and likelihood of reporting accurate information about natural history and reporting gibbon folktales was predicted only by reserve. No respondents from Jiaxi reported accurate information about natural history, but respondents from Diaoluoshan, Jianfengling, Limushan and Wuzhishan were all significantly more likely to report accurate information about natural history compared with respondents from Bawangling (Diaoluoshan: s.e. = 1.160, *z* = 2.327, *p* = 0.020; Jianfengling: s.e. = 1.289, *z* = 2.214, *p* = 0.027; Limushan: s.e. = 1.141, *z* = 2.052, *p* = 0.040; Wuzhishan: s.e. = 1.122, *z* = 2.679, *p* = 0.007). No respondents from Limushan reported any folktales, and respondents from Diaoluoshan, Jianfengling and Wuzhishan were significantly less likely to recount folktales compared with respondents from Bawangling (Diaoluoshan: s.e. = 1.191, *z* = −3.004, *p* = 0.003; Jianfengling: s.e. = 1.498, *z* = −2.909, *p* = 0.004; Wuzhishan: s.e. = 1.457, *z* = −3.882, *p* = 0.0001). A small number of respondents in each of these three reserves reported folktales from one of the two commonest folktale types collected during this survey (Diaoluoshan: folktale type 2, *n* = 4; Jianfengling: folktale type 1B, *n* = 1; Wuzhishan: folktale type 1, *n* = 1), although only one of these respondents (from Jianfengling) gave a detailed story, in contrast to 15 respondents from other landscapes (table 3). There was no statistical difference in levels of folktale reporting between Bawangling and Jiaxi or Yinggeling, but a small number of respondents from Jiaxi and Yinggeling (*n* = 3) recounted stories in folktale type 2b that clearly refer to monkeys instead of gibbons based on specific narrative details (e.g. presence of tail or red backside; description of crop-raiding), whereas all folktales reported from Bawangling refer to gibbons (table 3).

Gibbon-related folktales were reported by both Li respondents (*n* = 30) and Miao respondents (*n* = 7). Only folktale type 2 was reported by respondents from both ethnic backgrounds. Types 1, 3, 5 and 6 were reported exclusively by Li respondents, whereas type 4 was reported exclusively by Miao respondents.

## 4. Discussion

### 4.1. Gibbon traditional ecological knowledge on Hainan

The number of respondents who reported any gibbon-specific TEK was reasonably low compared with the total number interviewed for this study (*n* = 99/709). However, this TEK dataset still contains a diverse series of different types of indigenous knowledge about gibbons, including information on natural history, methods of exploitation and utilization, attitudes and values, and a series of different folktales, which were generally all recounted by older community members. Previous community-based awareness-raising and outreach activities to support gibbon conservation on Hainan have not included information on species-specific TEK [32,33,41], and so we are confident that our dataset represents previously unreported indigenous knowledge about the Hainan gibbon rather than knowledge gained by respondents through recent channels. The folktales and associated TEK on gibbons that we collected in this study represent an important extension to the existing body of folklore associated with gibbons and other apes that has been documented from other indigenous communities [28,51–54], and also to the diversity of folktales previously recorded from China [50,55] and wider animal-related folklore across Asia [56].

The almost complete difference in content between our newly collected TEK and historically recorded cultural practices and traditions about gibbons might indicate that TEK on gibbons, and maybe also other species, is surprisingly dynamic through time, a finding that has potentially important implications for the use of TEK about threatened species in adaptive management [6]. This possibility is supported by the fact that folktale type 1C is specifically reported to take place around the time of the Communist Liberation of Hainan in 1950 (table 3), indicating a historically recent modification of the ‘people turn into gibbons' folktale. However, it is also possible that at least some of the information about gibbons in Hainanese historical gazetteers might actually represent non-local lore, as these documents typically served as handover documents for civil servants assigned from elsewhere in China, and often explicitly reference well-known contemporary poems or books relating to mainland China when referring to environmental resources [35]. Indeed, some of the ‘fantastic' information on gibbon natural history recorded in Hainanese historical gazetteers and absent from current-day gibbon TEK on Hainan, including the accounts of ‘same-length-armed apes' and ‘stone apes', is recorded widely across China during the Imperial period [29,57,58].

Classification of Hainanese gibbon folktales within the standard Aarne–Thompson or Aarne-Thompson–Uther systems [59] is not straightforward due to the Western European bias of these classification systems, and previous incorporation of Chinese referents into this system has been criticized for uncritically adopting a European focus and supporting the false conclusion that the majority of the world's folktales are thematically related to European tales and that oikotypes are rare [60]. Identification of potential parallels is further limited by the general lack of distinction between monkeys, gibbons and other primates in most Western-influenced folklore classifications, whereas gibbons and monkeys are known to have distinct cultural associations in Chinese folklore [28]. The importance of social or historical context in shaping folktales [61–63], and of relating ‘tales to the art of tale telling and to the context in which it takes place’ ([64], p. 15), has long been recognized, although positioning them unquestioningly in specific and datable contexts is rarely possible. Folktales involving hunger and transmutation, such as in folktale type 1A, might originate from real famine events [65], which indigenous communities in the interior of Hainan are known to have experienced periodically into the twentieth century [66]. Indeed, one of our respondents recounted that in the past, local people had wished they were gibbons, because gibbons just needed fruit to eat. The sympathetic/positive depiction of gibbons in folktale types 1A and 4, and the idea of transformation between gibbons and humans in the commonest folktale types that we collected (types 1 and 2), is also similar to the regular historical depiction in mainland China of gibbons as virtuous or noble humans who were eventually transformed into supernatural beings [28]. Such transformations are probably all derived conceptually from the overall morphological similarity between humans and gibbons compared with other animals, and folktale type 2 (‘gibbons turned into people', with gibbons specifically referred to as ‘ancestors’) appears to constitute a Li creation myth. Similar myths of humans originating from monkeys have previously been recorded from Miao communities in China [55]. Although the specific folktales reported here are largely distinct from other indigenous stories involving gibbons, some narrative elements are common to stories from other Asian traditions, notably the association between gibbons and children, which probably reflects their shared relatively small body size and because both children and gibbons make crying noises or calls [52].

Indigenous communities around protected forest landscapes on Hainan consist primarily of two ethnic minorities, Li and Miao. Our analysis was unable to detect any statistical effect of ethnicity on overall patterns of reported TEK. However, respondents from different indigenous groups reported largely distinct gibbon folktale types. Some of this variation could reflect a sampling artefact associated with the small number of Miao respondents in our study who reported any folktales, which reflects the lower number of Miao respondents present in communities across Hainan [32]. However, we consider it likely that folktale type 4 (gibbons have helped/saved people) is indeed culturally restricted to Miao folklore, as it was never reported by the much larger number of Li respondents in our study (*n* = 598). Differences between Li and Miao interactions with their environments have been reported in previous studies, with Miao communities on Hainan historically more proficient at hunting gibbons [32]. Differences in indigenous environmental knowledge between respondents with different cultural backgrounds, despite long-term coexistence within the same landscapes, have also been demonstrated in other social–ecological systems [45,67].

### 4.2. Conservation implications of gibbon traditional ecological knowledge and its erosion

In the context of global-scale erosion of the ethnosphere, there is an urgent need to understand indigenous folklore about threatened species, and how this body of knowledge can contribute both to revitalization of biocultural diversity and to species-specific conservation management initiatives. In particular, for communities around Bawangling, the last remaining protected area on Hainan to contain gibbons, we anticipate that wider recognition and dissemination of existing indigenous gibbon folktales (e.g. through targeted awareness-raising activities within low-income subsistence communities adjacent to the protected area) can be used to strengthen emotional connections and values with the landscape and to promote ‘cultural ownership' of this critically endangered species together with increased local participation and support for conservation activities [6,13]. Such outreach activities should also be sensitive to the differences observed in this study in cultural traditions associated with gibbons between the different ethnic groups that live around Bawangling [32,33,41].

The relatively small number of respondents who reported any gibbon-specific TEK is consistent with an overall loss of TEK in Hainanese indigenous communities following the precipitous recent historical decline of the Hainan gibbon's total population. However, although gibbon-specific TEK was retained across all of the landscapes that we surveyed, we were able to identify statistically significant differences in TEK content between landscapes with different histories of gibbon survival or extinction. More respondents reported gibbon-related folktales from Bawangling (still contains gibbons) and from two of the three landscapes that have only recently lost gibbons, compared with Limushan (recently lost gibbons) and all landscapes from which gibbons have been absent for several decades. A completely opposite pattern is shown by accurate natural history information on gibbons, with more respondents reporting this component of TEK instead at Limushan and landscapes from which gibbons have been absent for several decades.

Although increased conservation education activities focusing on gibbons and other threatened biodiversity have been conducted at Bawangling and Yinggeling [32,33,41,68], spatial variation in past community-based conservation engagement is unlikely to be responsible for observed spatial variation in retention of different components of TEK as conservation activities have not incorporated specific information about local gibbon folklore, and wider awareness about gibbons in local communities around Bawangling is still relatively poor despite this outreach [38]. It is possible that this spatial variation in reported TEK could instead represent historical variation in TEK between landscapes. However, a small number of respondents from Diaoluoshan, Jianfengling and Wuzhishan still reported folktales representing the two commonest story types collected during our survey (although in all but one case providing only a brief description of story type without any detailed supporting narrative elements), indicating that these two folktales at least were formerly shared across the survey region. Some other aspects of gibbon-specific TEK collected from Hainan during this survey, such as the ecologically inaccurate idea that gibbons hold hands and form chains in order to work in relays to drink water, and the reported use of gibbon limb bones as chopsticks (table 2), are also a component of TEK in indigenous communities elsewhere in China rather than being spatially restricted to Hainan [28,29,69].

We therefore instead interpret these results as demonstrating spatial variation in erosion of different components of TEK associated with variation in local survival or extinction of gibbon populations. Our results therefore provide an interesting new example of shifting baseline syndrome, a phenomenon usually associated with perceptions of environmental conditions in the context of LEK rather than TEK, whereby a lack of intergenerational communication results in younger respondents having less knowledge about past biodiversity in systems that have experienced recent biological change [18,70,71]. In particular, our results demonstrate more rapid loss of folktales following gibbon extinction, in terms of both loss of story details and loss of overall stories, compared with other reported components of TEK. As no variation in overall retention of TEK by respondents was seen between landscapes, and most respondents in our study only volunteered a single piece of information about TEK, we interpret the corresponding increase in recounting of gibbon-specific natural history information from landscapes that have experienced older extinctions as a ‘compensatory response' from respondents who were no longer aware of gibbon folktales but still had something to report about gibbon TEK.

Few previous studies have investigated variation in vulnerability or resilience of different components of TEK in response to disruption of social–ecological systems, and provide limited information about the likely relative resilience of indigenous folktales in particular. Investigation of TEK about tuatara (*Sphenodon punctatus*) among Maori elders in New Zealand has shown that TEK on the species' cultural significance remained more common and detailed following tuatara decline in comparison to knowledge about the species' biology or ecology [72], suggesting that folktales might be expected to be relatively durable components of TEK compared with knowledge of natural history, the opposite pattern to what was observed in our study. Conversely, investigation of TEK loss in the Brazilian Amazon has demonstrated that medicinal and wild edible plant knowledge is more vulnerable than knowledge about firewood, canoe building and house building, potentially suggesting that comparable practical/utilitarian components of TEK might also be more resilient in other systems [73]. This finding matches our results that whereas gibbon-specific folktales have largely disappeared from landscapes where gibbons have been locally extinct for several decades, knowledge about how to hunt and use gibbons appears to be more resilient and shows no statistical drop-off with increased time since gibbon extinction.

This intriguing pattern of varying resilience of different components of TEK could be explained by the fact that older members of indigenous communities are typically the ‘cultural repositories’ of stories, traditions and worldviews [1,2,13], whereas knowledge about practical interactions with target species, such as hunting methods, might be shared more widely with younger community members when such species are regularly encountered in surrounding landscapes. Following the decline or extinction of such species, components of TEK that were originally shared with younger people might therefore be expected to persist for longer. However, this hypothesis might not account fully for our findings about varying resilience of different components of TEK, as anecdotal evidence from other social–ecological systems apparently suggests that practical knowledge about specific hunting practices can in some cases persist long beyond the disappearance of a target species and all community members who had hunted it. For example, Madagascar folktales of an ‘ogre' with the body of an animal but the face of a human, that could be rendered helpless on smooth rock outcrops because it was unable to move on flat surfaces, are suggested to be based on cultural memory of past hunting methods for the now-extinct sloth lemur (*Palaeopropithecus ingens*), which has probably been extinct for several centuries [23]. Similarly, Maori traditions collected during the nineteenth century about extinct moa (giant flightless birds), which probably died out over 500 years ago [74], consist largely of reported hunting methods [75,76]. Long-term persistence of utilitarian knowledge, in particular knowledge relating to hunting practices, may therefore be a more general pattern seen across indigenous cultures following extinction events even after the disappearance of other components of species-specific TEK.

In order to maintain biocultural diversity and identify mechanisms for linking conservation goals with indigenous worldviews, it is essential to develop a greater understanding not only about TEK on threatened species, but also about the dynamics of how such knowledge is lost. We therefore encourage further studies into patterns of variation in vulnerability and resilience of different components of TEK across different social–ecological systems, their duration following disruption to these systems, and the intrinsic or extrinsic factors that determine this variation. These questions have important wider conservation implications; for example, our study suggests that if folktales about possibly extinct species are present in local communities, does this mean that such species have only recently disappeared, or might even still survive? Through generating a firmer grounding about how the retention or erosion of TEK relates to the wider biocultural environment, this important tool will be able to provide even more unique new insights for informing conservation.

## Supplementary Material

Text-File S1

## Supplementary Material

Table S1

## Data accessibility

All data associated with this paper are available in the supplementary materials.

## References

[1] Berkes F 2012 Sacred ecology. Abingdon, UK: Routledge.

[2] Newing H 2011 Conducting research in conservation: a social science perspective. Abingdon, UK: Routledge.

[3] Reyes-García V, Martí N, McDade T, Tanner S, Vadez V 2007 Concepts and methods in studies measuring individual ethnobotanical knowledge. J. Ethnobiol. 27, 182–203. (doi:10.2993/0278-0771(2007)27[182:CAMISM]2.0.CO;2)

[4] Scientific Advisory Board of the UN Secretary-General. 2016 Policy brief: indigenous and local knowledge(s) and science(s) for sustainable development. See http://unesdoc.unesco.org/images/0024/002461/246104E.pdf (last accessed 4 April 2018).

[5] Berkes F, Folke C, Gadgil M 1995 Traditional ecological knowledge: biodiversity, resilience and sustainability. In Biodiversity conservation: problems and policies (eds Perring CA, Mäler KG, Folke C, Holling CS, Jansson BO), pp. 281–299. Dordrecht, The Netherlands: Kluwer.

[6] Berkes F, Colding J, Folke C 2000 Rediscovery of traditional ecological knowledge as adaptive management. Ecol. Appl. 10, 1251–1262. (doi:10.1890/1051-0761(2000)010[1251:ROTEKA]2.0.CO;2)

[7] Gómez-Baggethun E, Corbera E, Reyes-García V 2013 Traditional ecological knowledge and global environmental change: research findings and policy implications. Ecol. Soc. 18, 72 (doi:10.5751/ES-06288-180472) 26097492 10.5751/ES-06288-180472 PMC4471132

[8] Menzies CR 2006 Traditional ecological knowledge and natural resource management. Lincoln, NE: University of Nebraska Press.

[9] Colding J, Folke C 2001 Social taboos: ‘invisible’ systems of local resource management and biological conservation. Ecol. Appl. 11, 584–600.

[10] Bhagwat SA, Rutte C 2006 Sacred groves: potential for biodiversity management. Front. Ecol. Environ. 10, 519–524. (doi:10.1890/1540-9295(2006)4[519:SGPFBM]2.0.CO;2)

[11] Xu J, Ma ET, Tashi D, Fu Y, Lu Z, Melick D 2006 Integrating sacred knowledge for conservation: cultures and landscapes in southwest China. Ecol. Soc. 10, 7 (doi:10.5751/ES-01413-100207)

[12] Shen X, Li S, Wang D, Lu Z 2015 Viable contribution of Tibetan sacred mountains in southwestern China to forest conservation. Conserv. Biol. 29, 1518–1526. (doi:10.1111/cobi.12587) 26371613 10.1111/cobi.12587

[13] Fernández-Llamazares Á, Cabeza M In press Rediscovering the potential of indigenous storytelling for conservation practice. Conserv. Lett. (doi:10.1111/conl.12398)

[14] Lu F 2007 Integration into the market among indigenous peoples – a cross-cultural perspective from the Ecuadorian Amazon. Curr. Anthropol. 48, 593–602. (doi:10.1086/519806)

[15] Gómez-Baggethun E, Mingorría S, Reyes-García V, Calvet L, Montes C 2010 Traditional ecological knowledge trends in the transition to a market economy: empirical study in the Doñana natural areas. Conserv. Biol. 24, 721–729. (doi:10.1111/j.1523-1739.2009.01401.x) 20067484 10.1111/j.1523-1739.2009.01401.x

[16] Reyes-García V, Guèze M, Luz AC, Paneque-Gálvez J, Macía MJ, Orta-Martínez M, Pino J, Rubio-Campillo X 2013 Evidence of traditional knowledge loss among a contemporary indigenous society. Evol. Hum. Behav. 34, 249–257. (doi:10.1016/j.evolhumbehav.2013.03.002) 10.1016/j.evolhumbehav.2013.03.002 PMC3837211 24277979

[17] Tang R, McGavin MC 2016 A classification of threats to traditional ecological knowledge and conservation responses. Conserv. Soc. 14, 57–70. (doi:10.4103/0972-4923.182799)

[18] Turvey ST et al. 2010 Rapidly shifting baselines in Yangtze fishing communities and local memory of extinct species. Conserv. Biol. 24, 778–787. (doi:10.1111/j.1523-1739.2009.01395.x) 20067488 10.1111/j.1523-1739.2009.01395.x

[19] Lankford GE 1980 Pleistocene animals in folk memory. J. Am. Folklore 93, 293–304. (doi:10.2307/540573)

[20] Bauer AM, Russell AP 1985 *Hoplodactylus delcourti* n. sp. (Reptilia: Gekkonidae), the largest known gecko. NZ J. Zool. 13, 141–148. (doi:10.1080/03014223.1986.10422655)

[21] Miskelly CM 1987 The identity of the hakawai. Notornis 34, 95–116.

[22] Burney DA, Ramilisonina 1998 The kilopilopitsofy, kidoky, and bokyboky: accounts of strange animals from Belo-sur-mer, Madagascar, and the megafaunal ‘extinction window’. Am. Anthropol. 100, 1–10. (doi:10.1525/aa.1998.100.4.957)

[23] Godfrey LR, Jungers WL 2003 The extinct sloth lemurs of Madagascar. Evol. Anthropol. 12, 252–263. (doi:10.1002/evan.10123)

[24] Nunn PD, Reid NJ 2016 Aboriginal memories of inundation of the Australian coast dating from more than 7000 years ago. Aust. Geogr. 47, 11–47. (doi:10.1080/00049182.2015.1077539)

[25] Tehrani JJ 2013 The phylogeny of Little Red Riding Hood. PLoS ONE 8, e78871 (doi:10.1371/journal.pone.0078871) 24236061 10.1371/journal.pone.0078871 PMC3827309

[26] da Silva SG, Tehrani JJ 2016 Comparative phylogenetic analyses uncover the ancient roots of Indo-European folktales. R. Soc. open sci. 3, 150645 (doi:10.1098/rsos.150645) 26909191 10.1098/rsos.150645 PMC4736946

[27] Mittermeier RA, Rylands AB, Wilson DE 2013 Handbook of the mammals of the world, Vol. 3: *primates* Barcelona, Spain: Lynx Edicions.

[28] Van Gulik RH 1967 The gibbon in China: an essay in Chinese animal lore. Leiden, The Netherlands: EJ Brill.

[29] Geissmann T 2008 Gibbon paintings in China, Japan, and Korea: historical distribution, production rate and context. Gibbon J. 4, 1–38.

[30] Ye S, Heule F 2013 An evaluation of Robert van Gulik's *The Gibbon in China* and its place in modern Sinological discourse. Southeast Rev. Asian Stud. 35, 141–160.

[31] Turvey ST, Crees JJ, Di Fonzo MMI 2015 Historical data as a baseline for conservation: reconstructing long-term faunal extinction dynamics in Late Imperial–modern China. Proc. R. Soc. B 282, 20151299 (doi:10.1098/rspb.2015.1299) 10.1098/rspb.2015.1299 PMC4632630 26246553

[32] Chan BPL, Fellowes JR, Geissmann T, Zhang J 2005 Hainan gibbon status survey and conservation action plan. Hong Kong: Kadoorie Farm & Botanic Garden.

[33] Turvey ST, Traylor-Holzer K, Wong MHG, Bryant JV, Zeng X, Hong X, Long Y (eds). 2015 *International conservation planning workshop for the Hainan gibbon: final report*. London: Zoological Society of London.

[34] Bryant JV, Brulé A, Wong MHG, Hong X, Zhou Z, Han W, Jeffree TE, Turvey ST 2016 Detection of a new Hainan gibbon (*Nomascus hainanus*) group using acoustic call playback. Int. J. Primatol. 37, 534–547. (doi:10.1007/s10764-016-9919-8)

[35] Hong S 2003 Hainan difangzhi congkan. Haikou, China: Hainan Publishing House.

[36] Liu Z, Yu S, Yuan X 1984 The resource of the Hainan black gibbons at its present situation. Chin. Wildl. 6, 1–4.

[37] Zhou J, Wei F, Li M, Zhang J, Wang D, Pan R 2005 Hainan black-crested gibbon is headed for extinction. Int. J. Primatol. 26, 453–465. (doi:10.1007/s10764-005-2933-x)

[38] Turvey ST et al. 2017 How many remnant gibbon populations are left on Hainan? Testing the use of local ecological knowledge to detect cryptic threatened primates. Am. J. Primatol. 79, e22593 (doi:10.1002/ajp.22593) 10.1002/ajp.22593 27643665

[39] Kadoorie Farm & Botanic Garden. 2001–2003 Rapid biodiversity assessments in Hainan. Hong Kong: Kadoorie Farm & Botanic Garden.

[40] Smith AT, Xie Y 2008 A guide to the mammals of China. Princeton, NJ: Princeton University Press.

[41] Fauna & Flora International China Programme 2005 Action plan for implementing co-management in the Bawangling nature reserve and adjacent communities in Qingsong township. Beijing, China: Fauna & Flora International China Programme.

[42] Guest G 2006 How many interviews are enough? An experiment with data saturation and variability. Field Methods 18, 59–82. (doi:10.1177/1525822X05279903)

[43] Lau MWN, Fellowes JR, Chan BPL 2010 Carnivores (Mammalia: Carnivora) in South China: a status review with notes on the commercial trade. Mammal Rev. 40, 247–292. (doi:10.1111/j.1365-2907.2010.00163.x)

[44] White PCL, Jennings NV, Renwick AR, Barker NHL 2005 Questionnaires in ecology: a review of past use and recommendations for best practice. J. Appl. Ecol. 42, 421–430. (doi:10.1111/j.1365-2664.2005.01032.x)

[45] Turvey ST, Fernández-Secades C, Nuñez-Miño JM, Hart T, Martinez P, Brocca JL, Young RP 2014 Is local ecological knowledge a useful conservation tool for small mammals in a Caribbean multicultural landscape? Biol. Conserv. 169, 189–197. (doi:10.1016/j.biocon.2013.11.018)

[46] Turvey ST et al. 2015 Interview-based sighting histories can inform regional conservation prioritization for highly threatened cryptic species. J. Appl. Ecol. 52, 422–433. (doi:10.1111/1365-2664.12382) 25926709 10.1111/1365-2664.12382 PMC4407913

[47] Nash HC, Wong MHG, Turvey ST 2016 Determining status and threats of the Critically Endangered Chinese pangolin (*Manis pentadactyla*) in Hainan, China, using local ecological knowledge. Biol. Conserv. 196, 189–195. (doi:10.1016/j.biocon.2016.02.025)

[48] R Development Core Team. 2015 R: a language and environment for statistical computing. Vienna, Austria: R Foundation for Statistical Computing.

[49] Crawley MJ 2007 The R book. Chichester, UK: Wiley.

[50] Ting N 1978 A type index of Chinese folktales. Helsinki, Finland: Suomalainen Tiedeakatemia.

[51] Forth G 1988 Apes and dugongs: common mythological themes in diverse Southeast Asian communities. Contrib. SE Asian Ethnogr. 7, 189–229.

[52] Forth G 2014 Gugu: evidence from folk zoological nomenclature and classification for a mystery primate in southern Sumatra. Anthropos 109, 149–160.

[53] DeBuys W 2015 The last unicorn: a search for one of earth’s rarest creatures. New York, NY: Back Bay.

[54] Etiendem DN, Hens L, Pereboom Z 2011 Traditional knowledge systems and the conservation of Cross River gorillas: a case study of Bechati, Fossimondi, Besali, Cameroon. Ecol. Soc. 16, 22 (doi:10.5751/ES-04182-160322)

[55] Werner ETC 1922 Myths and legends of China. London, UK: Harrap.

[56] Newman P 2012 Tracking the weretiger: supernatural man-eaters of India, China and Southeast Asia. Jefferson, NC: McFarland.

[57] Wen R 2009 The distributions and changes of rare wild animals in China. Chongqing, China: Chongqing Science and Technology Press.

[58] Nappi C 2009 The monkey and the inkpot: natural history and its transformations in early modern China. Cambridge, MA: Harvard University Press.

[59] Uther H-J 2004 The types of international folktales: a classification and bibliography. Based on the system of Antti Aarne and Stith Thompson. Helsinki, Finland: Suomalainen Tiedeakatemia.

[60] Uther H-J 1996 Type- and motif-indexes 1980-1995: an inventory. Asian Folkl. Stud. 55, 299–317. (doi:10.2307/1178824)

[61] Zipes J 1983 The trials and tribulations of Little Red Riding Hood: versions of the tale in a sociocultural context. South Hadley, MA: Beregin & Garvey.

[62] Warner M 1995 From the beast to the blonde: on fairy tales and their tellers. London, UK: Random House.

[63] Tatar M 2003 The hard facts of the Grimms’ fairy tales. Princeton, NJ: Princeton University Press.

[64] Darnton R 1985 The great cat massacre and other episodes in French cultural history. New York, NY: Vintage.

[65] Zipes J 2002 Breaking the magic spell: radical theories of folk and fairy tales. *Revised edn* Lexington, KY: University Press of Kentucky.

[66] McClure FA 1922 Notes on the island of Hainan. Lingnaam Agric. Rev. 1, 66–79.

[67] Nyhus PJ, Sumianto, Tilson R 2003 Wildlife knowledge among migrants in southern Sumatra, Indonesia: implications for conservation. Environ. Conserv. 30, 192–199. (doi:10.1017/S0376892903000183)

[68] Kadoorie Farm & Botanic Garden. 2017 Programmes: Yinggeling National Nature Reserve, Hainan See http://www.kfbg.org/eng/yinggeling.aspx (accessed 1 December 2017).

[69] Fan P, Huo S 2009 Northern white-cheeked gibbon (*Nomascus leucogenys*) is on the edge of extinction in China. Gibbon J. 5, 1–9.

[70] Papworth SK, Rist J, Coad L, Milner-Gulland EJ 2009 Evidence for shifting baseline syndrome in conservation. Conserv. Lett. 2, 93–100. (doi:10.1111/j.1755-263X.2009.00049.x)

[71] Fernández-Llamazares Á, Díaz-Reviriego I, Luz AC, Cabeza M, Pyhälä A, Reyes-García V 2015 Rapid ecosystem change challenges the adaptive capacity of local environmental knowledge. Glob. Environ. Change 31, 272–284. (doi:10.1016/j.gloenvcha.2015.02.001) 26097291 10.1016/j.gloenvcha.2015.02.001 PMC4471143

[72] Ramstad KA, Nelson NJ, Paine G, Beech D, Paul A, Paul P, Allendorf FW, Daugherty CH 2007 Species and cultural conservation in New Zealand: Maori traditional ecological knowledge of tuatara. Conserv. Biol. 21, 455–464. (doi:10.1111/j.1523-1739.2006.00620.x) 17391195 10.1111/j.1523-1739.2006.00620.x

[73] Reyes-García V, Luz AC, Gueze M, Paneque-Gálvez J, Macía MJ, Orta-Martínez M, Pino J, TAPS Bolivian Study Team. 2013 Secular trends on traditional ecological knowledge: an analysis of different domains of knowledge among Tsimane’ men. Learn Individ. Differ. 27, 206–212. (doi:10.1016/j.lindif.2013.01.011) 24518817 10.1016/j.lindif.2013.01.011 PMC3837206

[74] Holdaway RN, Allentoft ME, Jacomb C, Oskam CL, Beavan NR, Bunce M 2014 An extremely low-density human population exterminated New Zealand moa. Nat. Comm. 5, 1–8. (doi:10.1038/ncomms6436) 10.1038/ncomms6436 25378020

[75] Archey G 1941 The moa: a study of the Dinornithiformes. Auckland, New Zealand: Unity Press.

[76] Anderson A 1989 Prodigious birds: moas and moa-hunting in prehistoric New Zealand. Cambridge, UK: Cambridge University Press.

