# Supplementary material for: Differential loss of components of traditional ecological knowledge following a primate extinction event
Source: R Soc Open Sci. 2018 Jun 13;5(6):172352. doi: 10.1098/rsos.172352 (PMC6030281; doi:10.1098/rsos.172352)
Supplement: Text-File S1 [file rsos172352supp1.docx]

**Text-File 1. Hainan wildlife community questionnaire**

**English-language version**

Date: _____________________________    Interviewer: _______________________________

Protected area: ______________________ Town / village: _____________________________

We are researchers from the Zoological Society of London. We want to know more about the environment and the forests around here, so I hope you can provide some information to help us better understand the local plants and animals, and any environmental changes that have taken place. The survey is anonymous and all the information you provide will only be used for research and analysis – we will not disclose any of your details to a third party.

1. Are you willing to participate in this survey? Yes   □   Unwilling   □

I hope you can try to answer all the questions because this survey is very important.

2. Age________  3. Gender________ 4. Ethnicity ______   5. Occupation ____________

We will now show you some photos of animals to look at. If you know or don’t know what they are, either answer is fine.

6. (Wild pig photo) Do you know what this animal is? Know / do not know

Know the name _____________ size / diet / appearance __________________________________________

If do not know, ask whether they have heard of this species and its features, and if they know the characteristics of this species, then continue to ask the following question __________________________________________

7. Have you seen these animals in the forest? Yes / No

If yes, the last time seen ____________________ and the place ________________________________

If seen, the total number of sightings ___________________ and frequency of sightings ______________

If no, then how do you know about this animal? ___________________________________________________

8. Do you think this animal is now: none / rare / not many / many

9. (Macaque photo) Do you know what this animal is? Know / do not know

Know the name _____________ size / diet / appearance __________________________________________

If do not know, ask whether they have heard of this species and its features, and if they know the characteristics of this species, then continue to ask the following question __________________________________________

10. Have you seen these animals in the forest? Yes / No

If yes, the last time seen ____________________ and the place ________________________________

If seen, the total number of sightings ___________________ and frequency of sightings ______________

If no, then how do you know about this animal? ___________________________________________________

11. Do you think this animal is now: none / rare / not many / many

12. (Gibbon photo) Do you know what this animal is? Know / do not know

Know the name _____________ size / diet / appearance __________________________________________

If do not know, ask whether they have heard of this species and its features, and if they know the characteristics of this species, then continue to ask the following question __________________________________________

13. Have you seen these animals in the forest? Yes / No

If yes, the last time seen ____________________ and the place ________________________________

If seen, the total number of sightings ___________________ and frequency of sightings ______________

If no, then how do you know about this animal? ___________________________________________________

14. Have you heard any stories about gibbons or anything else about them, such as uses? __________________________________________________________________________________________

15. Do you know any characteristics of gibbons (ecology, reproduction, behaviour, food)? __________________________________________________________________________________________

16. Do you know if other people have seen gibbons (time, place_______________________________________

Please provide the person's contact information ___________________________________________________

17. Do you know someone who has heard gibbons (time, place)? _____________________________________

18. Do you think this animal is now: none / rare / not many / many

19. Do you know anyone in the local area who has seen a gibbon within the past 10-15 years?

If yes, who ___________ location and any other information ___________________________________

* Can you take me to them, or call them?

20. (Clouded leopard photo) Do you know what this animal is? Know / do not know

Know the name _____________ size / diet / appearance __________________________________________

If do not know, ask whether they have heard of this species and its features, and if they know the characteristics of this species, then continue to ask the following question __________________________________________

21. Have you seen these animals in the forest? Yes / No

If yes, the last time seen ____________________ and the place ________________________________

If seen, the total number of sightings ___________________ and frequency of sightings ______________

If no, then how do you know about this animal? ___________________________________________________

22. Do you think this animal is now: none / rare / not many / many

23. (Bear photo) Do you know what this animal is? Know / do not know

Know the name _____________ size / diet / appearance __________________________________________

If do not know, ask whether they have heard of this species and its features, and if they know the characteristics of this species, then continue to ask the following question __________________________________________

24. Have you seen these animals in the forest? Yes / No

If yes, the last time seen ____________________ and the place ________________________________

If seen, the total number of sightings ___________________ and frequency of sightings ______________

If no, then how do you know about this animal? ___________________________________________________

25. Have you seen tracks/traces of a bear in the forest? (scratches, faeces, footprints, etc.) Yes / No

If yes, where, trail description, why do you think it was a bear, time, place _____________________________

26. Do you think this animal is now: none / rare / not many / many

27. (Pangolin photo) Do you know what this animal is? Know / do not know

Know the name _____________ size / diet / appearance __________________________________________

If do not know, ask whether they have heard of this species and its features, and if they know the characteristics of this species, then continue to ask the following question __________________________________________

28. Have you seen these animals in the forest? Yes / No

If yes, the last time seen ____________________ and the place ________________________________

If seen, the total number of sightings ___________________ and frequency of sightings ______________

If no, then how do you know about this animal? ___________________________________________________

29. Do you think this animal is now: none / rare / not many / many

30. Has anyone poached pangolins? Yes / No

       Are they local or foreign? Local / foreign

Was it for local use or to sell elsewhere? Local / foreign Place ______________________________

31. Has the poaching frequency changed? Yes / No

If yes, how / when / why the change? _______________________________________________________

32. (Binturong photo) Do you know what this animal is? Know / do not know

Know the name _____________ size / diet / appearance __________________________________________

If do not know, ask whether they have heard of this species and its features, and if they know the characteristics of this species, then continue to ask the following question __________________________________________

33. Have you seen these animals in the forest? Yes / No

If yes, the last time seen ____________________ and the place ________________________________

If seen, the total number of sightings ___________________ and frequency of sightings ______________

If no, then how do you know about this animal? ___________________________________________________

34. Do you think this animal is now: none / rare / not many / many

35. (Sambar photo) Do you know what this animal is? Know / do not know

Know the name _____________ size / diet / appearance __________________________________________

If do not know, ask whether they have heard of this species and its features, and if they know the characteristics of this species, then continue to ask the following question __________________________________________

36. Have you seen these animals in the forest? Yes / No

If yes, the last time seen ____________________ and the place ________________________________

If seen, the total number of sightings ___________________ and frequency of sightings ______________

If no, then how do you know about this animal? ___________________________________________________

37. Do you think this animal is now: none / rare / not many / many

38. (Giant anteater photo) Do you know what this animal is? Know / do not know

Know the name _____________ size / diet / appearance __________________________________________

If do not know, ask whether they have heard of this species and its features, and if they know the characteristics of this species, then continue to ask the following question __________________________________________

39. Have you seen these animals in the forest? Yes / No

If yes, the last time seen ____________________ and the place ________________________________

If seen, the total number of sightings ___________________ and frequency of sightings ______________

If no, then how do you know about this animal? ___________________________________________________

40. Do you think this animal is now: none / rare / not many / many

41. Do you think the number of any of these animals has decreased? Yes / No

      If yes, which animals, reduced by how much? _________________________________________________

42. Have any other animals around here also decreased in number? ____________________________________

43. What do you think is the rarest animal around here? ____________________________________________

44. Have any animals around here not decreased in number? ________________________________________

45. In addition to these animals, are there any animals that used to occur around here in the past but have now disappeared? Yes/ No

If yes, describe the animals, time and place seen or other source of news, and when they disappeared _________

46. Have you heard from elderly people that any other types of animals used to occur around here, but disappeared long ago? _______________________________________________________________________

47. Have you always lived in this village? Yes / No

If No, where did you live before? _______________________________________________________________

48. How often do you go into the forest (monthly or weekly)? ________________________________________

If you no longer enter the forest, what is the reason? ________________________________________________

What would you like to be allowed to do in the forest? ______________________________________________

49. Where do you usually go to get wood (area and distance)? ________________________________________

50. Did you used to go into the forest more in the past (time and frequency)? ____________________________

51. How much of your income comes from things collected in the forest? _______________________________

(Play five kinds of animal sounds)

52. Chimpanzee (____): Have you heard it? Yes/ rarely / not many / many / none Name? ______________

How many times? _________ When? _____________ Place? ___________________________________

53. Gibbon (____): Have you heard it? Yes/ rarely / not many / many / none Name? _________________

How many times? _________ When? _____________ Place? ___________________________________

54. Peafowl (____): Have you heard it? Yes/ rarely / not many / many / none Name? _______________

How many times? _________ When? _____________ Place? __________________________________

55. Howler monkey (____): Have you heard it? Yes/ rarely / not many / many / none Name? ____________

How many times? _________ When? _____________ Place? __________________________________

56. Screaming piha (____): Have you heard it? Yes/ rarely / not many / many / none Name? ___________

How many times? _________ When? _____________ Place? __________________________________

Do you know anyone in the village who is familiar with the wild animals here? Can you help us find them?

**Chinese-language version**

海南野生动物社区调查问卷

日期:_____________________________ 调查员: ____________________________

保护区: __________________________ 乡/村: ______________________________

我们是伦敦动物学会的调查员。我们想要多了解这里周围的环境与林子，所以希望你能提供一些相关信息，帮助我们更了解以前山上的动植物以及多年来的环境变迁。这个是个不记名调查，你提供的所有信息只会被用作研究分析，不会向第三方透露。

1. 你是否愿意参与这次问卷调查？ 愿意 □ 不愿意 □

希望你可以尽量回答我们这份卷子的所有问题，这个对我们调查非常重要。

2. 年龄________ 3. 性别_______ 4. 民族____________ 5. 职业____________

现在给你们看一些动物的照片，看你们认不认识，不认识也没有关系。

6. （wild pig photo）你知道这是什么动物吗？ 知道 / 不知道

知道，名称_____________大小/食性/外貌__________________________________________

不知道，问是否有听说过这物种以及它的特征，如果知道物种特征，继续问以下问题______________________________________________________________________________

7. 你在山上见过这动物吗? 有 / 没有

有，上次看见的时间_________________________________地点____________________

看见总次数_________________如果常见，频率是___________________________________

没有，你是如何知道这动物的？_______________________________________________

8. 你觉得这个动物现在是 没有 / 很少 / 不多 / 很多

9. （macaque photo）你知道这是什么动物吗？ 知道 / 不知道

知道，名称_____________大小/食性/外貌__________________________________________

不知道，问是否有听说过这物种以及它的特征，如果知道物种特征，继续问以下问题______________________________________________________________________________

10. 你在山上见过这动物吗? 有 / 没有

有，上次看见的时间_________________________________地点____________________

看见总次数_________________如果常见，频率是___________________________________

没有，你是如何知道这动物的？__________________________________________________

11. 你觉得这个动物现在是 没有 / 很少 / 不多 / 很多

12. （gibbon photo）你知道这是什么动物吗？ 知道 / 不知道

知道，名称_____________大小/食性/外貌__________________________________________

不知道，问是否有听说过这物种以及它的特征，如果知道物种特征，继续问以下问题

13. 你在山上见过这动物吗? 有 / 没有

有，上次看见的时间_________________________________地点____________________

看见总次数_________________如果常见，频率是___________________________________

没有，你是如何知道这动物的？__________________________________________________

14. 你听说过有关长臂猿的故事或事情（如用途）吗？___________________________________

15. 你知道长臂猿的特征吗（生态、繁殖、行为、食物）？_______________________________

16. 你知道其他人在山上见过这动物吗（时间、地点）? ________________________________ 可否提供这个人的联系方式？________________________________________________________

17. 你知道有人打过长臂猿吗（时间、地点）？_________________________________________

18. 你觉得这个动物现在是 没有 / 很少 / 不多 / 很多

19. 你可知道附近有人在近10-15年内有见过长臂猿吗?

有，谁___________什么时候____________地点______________其它信息_______________

*问是否能带我去找他或打给他?

20. （clouded leopard photo）你知道这是什么动物吗？ 知道 / 不知道

知道，名称_____________大小/食性/外貌__________________________________________

不知道，问是否有听说过这物种以及它的特征，如果知道物种特征，继续问以下问题

21. 你在山上见过这动物吗? 有 / 没有

有，上次看见的时间_________________________________地点____________________

看见总次数_________________如果常见，频率是___________________________________

没有，你是如何知道这动物的？__________________________________________________

22. 你觉得这个动物现在是 没有 / 很少 / 不多 / 很多

23. （bear photo）你知道这是什么动物吗？ 知道 / 不知道

知道，名称_____________大小/食性/外貌__________________________________________

不知道，问是否有听说过这物种以及它的特征，如果知道物种特征，继续问以下问题

24. 你在山上见过这动物吗? 有 / 没有

有，上次看见的时间_________________________________地点____________________

看见总次数_________________如果常见，频率是___________________________________

没有，你是如何知道这动物的？__________________________________________________

25. 你有没有在山上发现过熊留下的踪迹（抓痕、粪便、脚印等） 有 / 没有

有，踪迹的描述、为什么认为是熊的、时间、地点__________________________________

26. 你觉得这个动物现在是 没有 / 很少 / 不多 / 很多

27. （pangolin photo）你知道这是什么动物吗？ 知道 / 不知道

知道，名称_____________大小/食性/外貌__________________________________________

问是否有听说过这物种以及它的特征，如果知道物种特征，继续问以下问题______________________________________________________________________________

28. 你在山上见过这动物吗? 有 / 没有

有，上次看见的时间_________________________________地点____________________

看见总次数_________________如果常见，频率是___________________________________

没有，你是如何知道这动物的？__________________________________________________

29. 你觉得这个动物现在是 没有 / 很少 / 不多 / 很多

30. 这里有人抓穿山甲么? 有 / 没有

有，当地还是外来人？ 当地 / 外来

当地使用还是卖到外地？ 当地 / 外地，地方________________________________________

31. 捕猎频率有改变吗？ 有 / 没有 有，怎么/什么时候/为什么改变？_____________________

32. （binturong photo）你知道这是什么动物吗？ 知道 / 不知道

知道，名称_____________大小/食性/外貌__________________________________________

不知道，问是否有听说过这物种以及它的特征，如果知道物种特征，继续问以下问题

33. 你在山上见过这动物吗? 有 / 没有

有，上次看见的时间_________________________________地点____________________

看见总次数_________________如果常见，频率是___________________________________

没有，你是如何知道这动物的？__________________________________________________

34. 你觉得这个动物现在是 没有 / 很少 / 不多 / 很多

35.（sambar photo）你知道这是什么动物吗？ 知道 / 不知道

知道，名称_____________大小/食性/外貌__________________________________________

不知道，问是否有听说过这物种以及它的特征，如果知道物种特征，继续问以下问题

36. 你在山上见过这动物吗? 有 / 没有

有，上次看见的时间_________________________________地点____________________

看见总次数_________________如果常见，频率是___________________________________

没有，你是如何知道这动物的？__________________________________________________

37. 你觉得这个动物现在是 没有 / 很少 / 不多 / 很多

38. （giant anteater photo）你知道这是什么动物吗？ 知道 / 不知道

知道，名称_____________大小/食性/外貌__________________________________________

不知道，问是否有听说过这物种以及它的特征，如果知道物种特征，继续问以下问题

39. 你在山上见过这动物吗? 有 / 没有

有，上次看见的时间_________________________________地点____________________

看见总次数_________________如果常见，频率是___________________________________

没有，你是如何知道这动物的？__________________________________________________

40. 你觉得这个动物现在是 没有 / 很少 / 不多 / 很多

41. 你觉得以上这些动物的数量在这些年有没有减少？ 有 / 没有

有，什么动物，减少多少？___________________________________________________

42. 除了以上的动物，这里还有什么动物越来越少了？__________________________________

43. 你觉得这里现在最稀有的动物是那种？____________________________________________

44. 你觉得那种动物数量没有减少过？________________________________________________

45. 除了以上的动物，这里以前还有什么动物是现在没有的？ 有 / 没有

有，描述动物，见到的时间和地点，或者其它消息来源，什么时候消失的？____________ 有没有从老人听说附近有其它动物，但是在很久以前就已经消失了？__________________

47. 你是否一直住在这村子? 是 / 否，你住在这里多久_________以前住哪______________

48. 你一般多久会进山一次（每月或每周）？_______________________________________

如果现在已经不再上山，原因是什么？_________________________________________

你希望能允许上山么? ________________________________________________________

49. 通常会去哪片林子（片区与距离）？___________________________________________

50. 你进山的频率以前会更高吗（时间与频率）？___________________________________

51. 你经济收入的多大部分来自林子采来的东西?____________________________________

52. 第一段（____）：有听过吗? 有，很少 / 不多 / 很多/ 没有 名称________________________

几次_____时间______________________地点_______________________________________

53. 第二段（____）：有听过吗? 有，很少 / 不多 / 很多/ 没有 名称_______________________

几次_____时间______________________地点_______________________________________

54. 第三段（____）：有听过吗? 有，很少 / 不多 / 很多/ 没有 名称________________________

几次_____时间______________________地点_______________________________________

55. 第四段（____）：有听过吗? 有，很少 / 不多 / 很多/ 没有 名称_______________________

几次_____时间______________________地点_______________________________________

56. 第五段（____）：有听过吗? 有，很少 / 不多 / 很多/ 没有 名称_______________________

几次_____时间______________________地点_______________________________________

你可知道村子里有哪个人熟悉野生动物？ 能否协助我们找到他吗？
